# DECISION TO SCREEN: CAREGIVERS VOICES IN MAMMOGRAPHY DECISION-MAKING WITH DEMENTIA

**DOI:** 10.1093/geroni/igae098.4153

**Published:** 2024-12-31

**Authors:** Adele Crouch, Nicole Fowler

**Affiliations:** Indiana University, Indianapolis, Indiana, United States; Indiana University, Indianapolis, Indiana, United States

## Abstract

The USPSTF recommends mammograms through 74 years old and data suggest that individuals should have a life expectancy of 10 years for a mortality benefit. Mammography beyond 75 may increase burden and risk of overdiagnosis and overtreatment; dementia may exacerbate this burden. Despite guidelines and changing risks and benefits, many over 75 who have life limiting illnesses receive mammograms; it’s unclear what motivates these decisions. Therefore, this descriptive qualitative analysis explored dementia caregivers descriptions about the decision to undergo or not undergo mammography screening for their relatives with dementia. Sixty dyads from an IRB-approved study on mammography decision-making completed interviews. Regarding the decision to screen, a main theme emerged: unconscious choice. Sub-themes included: 1) automatic reoccurring event; 2) healthcare provider recommended; 3) hospital/healthcare organization recommended, 4) family history. Some identified it as an automatic reoccurring event (“It was needed for general health, and it’s something that we do in our household religiously.”). In consultation with a healthcare provider some decided to proceed with mammograms (“Her doctor said it was time for her annual mammogram and he would schedule it”). Numerous were prompted to receive a mammogram by a hospital/healthcare organization (“We received a reminder in the mail that it was time for me, and it was time for her, and I just made the appointment. There was no discussion.”). Lastly, participants identified that their family history of cancer prompted them. Overall, many participants may not have recognized mammography as a choice.

